# Risk Factors of Gout in MDR-TB Patients in Eritrea: A Case-Control Study

**DOI:** 10.1155/2019/9429213

**Published:** 2019-05-21

**Authors:** Mulugeta Russom, Hager Tesfaselassie, Rozina Goitom, Tadese Ghirmai, Freweini Weldedhawariat, Abiel Berhe, Dawit Tesfai, Merhawi Debesai, Tesfit Berhane, Henok G. Woldu

**Affiliations:** ^1^Eritrean Pharmacovigilance Centre, National Medicines and Food Administration, Asmara, Eritrea; ^2^School of Public Health, Asmara College of Health Sciences, Asmara, Eritrea; ^3^Department of Diagnostic Imaging, Orotta National Referral and Teaching Hospital, Asmara, Eritrea; ^4^School of Pharmacy, Asmara College of Health Sciences, Asmara, Eritrea; ^5^Biostatistics & Research Design Unit, School of Medicine, University of Missouri, Columbia, MO, USA

## Abstract

Though the incidence of gout in general population is less than 5% (globally), a study conducted in Eritrea among patients with multidrug resistant tuberculosis (MDR-TB) revealed a higher incidence (25%). Other similar studies conducted elsewhere, however, did not report gout as an adverse effect. It is unknown why a higher incidence of gout is being reported in Eritrea compared to similar studies from other countries. The objective of this study is therefore to explore risk factors for the increased risk of incident gout among MDR-TB patients in Eritrea. A case-control study was conducted in Merhano MDR-TB National Referral Hospital in Eritrea. All MDR-TB patients diagnosed with gout between June 2011 and June 2018 were considered as cases. Controls matched by age, sex, and cumulative drug exposure time were selected from the same study population (1:1 ratio). A total of 84 MDR-TB patients, 42 cases and 42 controls, were included in this study. No patient from the control group has comorbidities, while six patients from the case group have diabetes (*χ*2 = 6.46, df=1, p=0.026). Patients having tachycardia (OR=3.26, 95% CI=1.28, 8.27), alopecia (OR=3.11, 95% CI=1.00, 9.67), and gastrointestinal upset (OR=3.17, 95% CI=1.26, 7.96) as adverse effects and being on prolonged use of propranolol (OR=3.26, 95% CI=1.28, 8.27) were found to be more likely to develop incident gout compared to their controls. In conclusion, MDR-TB patients with diabetes mellitus, tachycardia, alopecia, and gastrointestinal upset and on prolonged use of propranolol tablet had increased risk of incident gout.

## 1. Introduction

Multidrug resistant tuberculosis (MDR-TB) is defined as a disease caused by mycobacterium tuberculosis (TB) that is resistant to at least isoniazid and rifampicin [1]. It is one of the major public health problems that hampers the control of tuberculosis (TB) globally. Patients with MDR-TB are required to take several second-line anti-TB drugs for about two years. The long duration of exposure to treatment and diverse drugs prescribed increases the risk of adverse drug reactions (ADRs) that could negatively affect treatment adherence and consequently treatment success rate [2, 3].

Though globally the incidence of gout in the general population is less than 5% [4], a study conducted in Eritrea revealed higher incidence of gout (25%) following use of second-line anti-TB drugs some of which resulted treatment interruptions in several patients [5]. Other similar studies conducted elsewhere, however, did not report gout as an adverse effect [6–15]. Gout is a painful rheumatic disease characterized by deposition of uric acid crystals in the joints and elevation of serum uric acid levels, leading to acute inflammatory arthritis [16, 17]. It is unknown why a higher incidence of gout is being reported in Eritrea compared to other studies reported elsewhere. The authors, however, speculated that this adverse effect could be linked to the second-line antituberculosis drugs since the patients had no history of gout before the initiation of MDR-TB treatment.

As hypothyroidism was one of the frequently reported ADRs among MDR-TB patients in Eritrea, there was an assumption that it could partly explain the exaggerated incident gout. This is because hypothyroidism has been reported as a risk factor for gout and/or hyperuricemia in non-MDR-TB population [18–21]. There might also be other risk factors, other than second-line anti-TB drugs, that could contribute to development of gout. The objective of this study was therefore to identify possible risk factors for the high incidence of gout in MDR-TB patients in Eritrea.

## 2. Methods

### 2.1. Study Design and Setting

This case-control study was conducted in Merhano MDR-TB National Referral Hospital which is the only hospital in Eritrea for treatment initiation, hospitalization, and follow-up of MDR-TB patients. It is a fifty-bed hospital established in June 2011 in Merhano, Southwest of Asmara.

### 2.2. Data Source

Historical longitudinal data was acquired from registers, medical or clinical cards, and laboratory records of the MDR-TB patients documented between June 2011 and June 2018. A structured questionnaire was developed to assess the medical records. Patients' background characteristics, drug reaction and drug details, baseline, and follow-up laboratory results were extracted from the clinical records. Data was also collected in October 2018.

### 2.3. Study Population

#### 2.3.1. Selection of Cases

All MDR-TB patients diagnosed with gout between June 2011 and June 2018, who had normal baseline data while being admitted to the hospital were considered as cases. Patients having gout, arthritis, rheumatoid arthritis, osteoarthritis, hypothyroidism, and/or HIV/AIDS during admission to the hospital were excluded from the study to reduce outcome/exposure misclassification bias.

#### 2.3.2. Selection of Controls

Controls were those who did not develop gout while in second-line antituberculosis drugs during the aforementioned time-frame. They were selected from the same study population as the cases. Controls matched by age, sex, and cumulative drug exposure time were selected from Merhano MDR-TB hospital registry without knowing their status. For every case, one matched control was chosen. Exclusion and inclusion criteria set for the cases were applied to the controls. Moreover, the procedure used to validate cases was applied to controls.

### 2.4. Exposure Definition

MDR-TB treatment is a two-phase process. There are four oral second-line drugs, namely, Levofloxacin, Ethionamide, Pyrazinamide, and Cycloserine. These drugs were taken for about 24 months; at least eight months on intensive; and 12 months on continuation phase. The fifth was an injectable drug, Kanamycin, which was taken only for six months while patients were admitted in hospital. Vitamin B6 (pyridoxine) was also prescribed to all patients as a supplement to prevent incidences of neurologic adverse events related to second-line anti-TB drugs. Daily dose was given according to their body weight as recommended by the WHO [22]. In addition to these drugs, a patient might have taken other drugs concomitantly to treat other comorbidities and alleviate some long-term adverse effects. In this study, possible risk factors for gout including other chronic medications, adverse effects that stayed long, and comorbidities were also considered as exposures.

### 2.5. Outcome Definition and Measurement

The primary outcome measure of this study was documented diagnosis of incident gout following initiation of second-line antituberculosis drugs. It means that patients should not had history of gout before their admission to the MDR-TB national referral hospital. Gout is defined as a painful rheumatic disease characterized by deposition of uric acid crystals in the joints and elevation of serum uric acid levels, leading to acute inflammatory arthritis [18, 19]. For this study, cases were classified as clinical gout based on Rome's gout classification criteria [23]. According to the classification criteria, patients with increased serum uric acid level (>7mg/dL for males and >6mg/dL for females) accompanied with joint pain and/or joint inflammation or swelling were identified as cases.

### 2.6. Statistical Analysis

As an initial step, exploratory analyses on different variables of interest were performed on the data to summarize and quantify it and investigate for important trends. Confirmatory analyses were then used to assess the statistical association between the outcome, gout, and several exploratory variables of interest. Chi-square goodness of fit test for categorical and two sample t-test for continuous variables were used in order to assess statistical associations.

To assess the main risk factors for gout among MDR-TB patients, bivariate and age adjusted multivariable logistic regression model were used. From the fitted model, odds ratios (ORs) and corresponding 95% confidence intervals were calculated. All p-values calculated were two-sided and p-values less than the significance level (alpha=0.05) were considered statistically significant. All statistical analyses were performed using SAS 9.4.

### 2.7. Ethical Considerations

Ethical clearance to conduct the study was obtained from the research ethics and protocol review committee of the Ministry of Health. All the data acquired was used only for the purposes of this study and is strictly kept confidential. 

## 3. Results and Discussion

### 3.1. Results

A total of 84 MDR-TB patients, 42 cases and 42 controls, were included in this study. The mean age and standard deviation (SD) of the case and control groups were 40.57 (13.78) and 39.43 (15.22), respectively. The average drug exposure time in months was 20 (SD = 6.12) for the case group and 19 (SD=7.44) for the control group with respective median number of drugs being 7.0 (range = 4-10) and 6.5 (range=6-9). None of the control group patients had a known comorbidity while six patients from the case group were found to be diabetic. To ensure the two groups were matched appropriately for the main variables of interest, chi-square test of association for categorical variables and two sample t-test for continuous variables were used. However, the groups showed a statistically significant difference in the presence of comorbidity (p-value=0.026). Among the cases, the average number of months to the onset of gout was 5.7 (SD =5.34) (Table 1).

Hypothyroidism was observed in 62% of the case group and 57% of the control group. GI upset, alopecia, and tachycardia as adverse drug reactions were reported twice higher in the case group than the control group (Table 2). Additionally, the case group reported tachycardia more than double the control group (81% vs 38%). The two groups did not show a statistically significant difference in the drug adverse reactions reported for hypothyroidism (p-value = 0.657), alopecia (p-value = 0.068), psychotic symptoms (p-value = 0.533), and insomnia (p-value = 0.212) (Table 2).

In addition to the regular MDR-TB medications, patients were taking other medications for other comorbidities and chronic conditions. Out of the total 42 MDR-TB patients in the case group, 23 were on propranolol while only 10 of the 42 patients from the control group were on propranolol. No patient from the control group was taking diabetes mellitus drugs, while six patients in the 158-case group were taking either insulin (5) or metformin (1) (Table 3).

Patients who had tachycardia (OR=3.10; 95% CI=1.24, 7.75) were three times more likely to develop gout compared to their controls. In the multivariate conditional logistic regression analysis, no statistically significant difference was observed between the case and control groups for hypothyroidism exposure (OR=1.31; 95% CI=0.47, 3.68) (Table 4). While alopecia, tachycardia, GI upset, and number of drugs appeared to be significantly associated with incident gout in MDR-TB patients, MDR-TB patients taking higher number of drugs had 83% increased risk of incident gout (Table 4).

## 4. Discussion

In this study, presence of tachycardia and prolonged use of propranolol, higher number of drugs, alopecia, GI upset, and diabetes were found to be possible risk factors for the high incidence of gout among MDR-TB patients in Eritrea.

Patients with tachycardia were about three times more likely to develop gout compared to the control group. Due to the fact that all patients who developed tachycardia while on second-line anti-TB drugs were taking propranolol (40mg once or twice daily for at least three months), incident gout could partially be attributed to the effect of this beta-blocker. This is because beta-blockers like propranolol are known to increase the amount of uric acid in blood [24–26], which is a predisposing factor for the development of gout. However, it is difficult to establish causation between tachycardia (through intake of propranolol) and gout from this study since it is unknown which event developed first.

Intake of higher number of drugs was identified as a risk factor for development of incident gout. This could be attributed to propranolol to which the case group was highly exposed compared to their controls. However, the effect of alopecia and GI upset in the development of incident gout could not be explained and thus requires further studies.

Diabetes mellitus, the only comorbidity in this study, was identified as a risk factor for gout, which is consistent with studies reported elsewhere [27]. This can be explained by the fact that serum uric acid is increased due to hyperinsulinemia as a consequence of insulin resistance by both reducing renal uric acid secretion [28] and accumulating substrates for uric acid production [29]. This, however, cannot rule out the effect of second-line anti-TB drugs like pyrazinamide and ethionamide in the development of gout in MDR-TB patients.

Initially, the hypothesis of the study was that hypothyroidism might be a major risk factor for the development of gout in MDR-TB patients as there is biological explanation for this association. The assumption was also triggered by the fact that hypothyroidism and gout were among the frequently reported adverse effects with the use of second-line anti-TB drugs in Eritrea. This study nonetheless did not find enough evidence to corroborate the initial hypothesis.

The mean time to onset of gout in MDR-TB patients in months was found to be short (5.7). This shows that majority of patients (71%) developed gout while they were on intensive phase of treatment or admitted to the MDR-TB referral hospital. This partially rules out the effect of differences in nutritional status among patients as they were taking the same diet during their stay in the hospital.

Absence of recall bias and applying consistent data collection process in the cases and controls are some of the strengths of the study. Moreover, the study protocol was developed using ENCePP checklist [30] and reported according to the STROBE guidelines [31]. The study was however not without limitations. Due to the nature of the study that used historical data, some independent variables that are associated with gout might not be fully addressed. As temporal association of tachycardia or intake of propranolol and gout is not clearly known, we cannot infer causation with these findings. Moreover, the diagnosis of gout was not confirmed as cases were identified based on Rome's clinical gout classification criteria [23]. The small sample size also limited our statistical power.

## 5. Conclusions

Prolonged use of propranolol, tachycardia, alopecia, GI upset, number of drugs taken, and diabetes mellitus were identified as possible risk factors for the development of gout. Thus, the high incidence of gout in MDR-TB patients could be related to second-line anti-TB drugs and the aforementioned risk factors. In addition to compromising quality of life of patients and treatment adherence, gout is a risk factor for hypertension, cardiovascular diseases, atherosclerosis, and metabolic syndrome. Hence, care should be exercised in preventing incident gout in MDR-TB patients. To minimize or prevent the occurrence of gout, patients with diabetes mellitus should be closely monitored. Moreover, the root cause of tachycardia among MDR-TB patients needs to be identified to determine the appropriateness of propranolol use in these patients. Its prolonged use should also be reconsidered.

## Figures and Tables

**Table 1:** Comparing the baseline characteristics of the case and control groups.

Variable Name	Cases	Controls	P-value
Total No. Patients	42	42	

Ethnicity			
Tigrigna	32	36	0.522^+^
Tigre	4	2	
Others	6	4	

Any Comorbidity			
No	36	42	0.026^+^
Yes	6	0	

Mean (SD)			
Age	40.57 (13.78)	39.43 (15.22)	0.719^++^
Weight	43.38 (9.55)	43.69 (11.24)	0.892^++^
Drug Exposure (Months)	20.33 (6.12)	19.43 (7.44)	0.544^++^
Number of Drugs	7.05 (1.03)	6.62 ( 0.73)	0.031^++^
Onset of gout	5.71 (5.34)		

Gout Phase (Median, Range)	1.0 (1 - 2)		

^+^The p-values were obtained from a chi-square goodness of fit test. For those with a cell count of zero, p-value was obtained after applying a continuity adjustment. ^++^The p-value was obtained from a two-sample t-test.

**Table 2:** Summary of major adverse drug reactions among MDR-TB patients and their association with incident gout.

Adverse Drug Reaction	Cases	Controls	P-value
Hypothyroidism			
Yes	26	24	0.657
No	16	18	

GI upset			
Yes	22	11	0.014
No	20	31	

Tachycardia			
Yes	22	11	0.014
No	20	31	

Alopecia			
Yes	13	6	0.068
No	29	36	

Psychotic symptoms			
Yes	7	5	0.533
No	35	37	

Insomnia			
Yes	8	4	0.212
No	34	38	

Other Symptoms			
Yes	9	14	0.213
No	33	28	

GI: gastrointestinal. P-value was obtained from Chi-square goodness of fit test.

**Table 3:** Effect of other drug exposures (other than second-line anti-TB drugs) on the development of gout in MDR-TB patients.

Drug Generic Name	Cases	Controls	P-value
Propranolol			
Yes	22	11	0.014
No	20	31	

Levothyroxine			
Yes	6	8	0.558
No	36	34	

Diazepam			
Yes	7	5	0.533
No	35	37	

Amitriptyline			
Yes	4	3	0.999++
No	38	39	

Insulin			
Yes	5	0	0.055++
No	37	42	

Metformin			
Yes	1	0	0.999++
No	41	42	

DM drugs			
Yes	6	0	0.026++
No	36	42	

++ P-value is obtained using Fishers exact chi-square test; DM: diabetes mellitus drugs.

**Table 4:** Bivariate and multivariable logistic model analyses for assessing the risk factors associated with the development of gout among MDR-TB patients.

	Bivariate Analysis		Multivariate Analysis	
	Estimate (SE)	Crude OR (95% CI)	Estimate (SE)	Adjusted OR (95% CI)
Hypothyroidism (Yes)	0.198 (0.445)	1.22 (0.51, 2.92)	0.20 (0.446)	1.22 (0.51, 2.94)
GI Upset (Yes)	1.131 (0.468)	3.10 (1.24, 7.75)	1.15 (0.471)	3.17 (1.26, 7.96)
Alopecia (Yes)	0.989 (0.553)	2.69 (0.91, 7.95)	1.13 (0.579)	3.11 (1.00, 9.67)
Tachycardia (Yes)	1.13 (0.477)	3.10 (1.24, 7.75)	1.18 (0.474)	3.26 (1.28, 8.27)
Number of Drugs	0.571 (0.277)	1.77 (1.03, 3.05)	0.61 (0.286)	1.83 (1.05, 3.21)

OR: odds ratio; SE: standard error; CI: confidence interval; GI: gastrointestinal.

## Data Availability

The data used to support the findings of this study are available from the corresponding author upon request.
